# Late-onset vascular complications of radiotherapy for primary brain tumors: a case–control and cross-sectional analysis

**DOI:** 10.1007/s11764-023-01350-z

**Published:** 2023-05-05

**Authors:** María-José Ibáñez-Juliá, Alberto Picca, Delphine Leclercq, Giulia Berzero, Julian Jacob, Loïc Feuvret, Charlotte Rosso, Cristina Birzu, Agusti Alentorn, Marc Sanson, Camille Tafani, Flavie Bompaire, Luis Bataller, Khê Hoang-Xuan, Jean-Yves Delattre, Dimitri Psimaras, Damien Ricard

**Affiliations:** 1 Department of Neurology Mazarin, Hôpitaux Universitaires Pitié-Salpêtrière Charles Foix. Assistance Publique Hôpitaux de Paris (APHP), Paris, France; 2 Department of Neurology, Ascires Biomedical Group, Valencia, Spain; 3 OncoNeuroTox Group: Center for Patients With Neurological Complications of Oncologic Treatments, Hôpitaux Universitaires Pitié-Salpêtrière Charles Foix, Hôpital d’Instruction Des Armées Percy, Paris, France; 4 Sorbonne Universités, Inserm, CNRS, UMR S 1127, Institut du Cerveau et de la Moelle épinière, HP Paris, France; 5 Department of Neuroradiology, Hôpitaux Universitaires Pitié-Salpêtrière Charles Foix. Assistance Publique Hôpitaux Paris (APHP), Paris, France; 6 Neurology Unit, IRCCS San Raffaele Scientific Institute, Milan, Italy; 7 Vita-Salute San Raffaele University, Milan, Italy; 8 Department of Radiotherapy, Hôpitaux Universitaires Pitié-Salpêtrière Charles Foix. Assistance Publique Hôpitaux de Paris (APHP), Paris, France; 9 Radiation Therapy Department, Hospices Civils de Lyon, Lyon, France; 10 Department of Vascular Neurology, Hôpitaux Universitaires Pitié-Salpêtrière Charles Foix. Assistance Publique Hôpitaux de Paris (APHP), Paris, France; 11 Department of Neurology, Hôpital d’Instruction Des Armées Percy, Service de Santé Des Armées, Clamart, France; 12 Neurology Department, Hospital Universitari i Politècnic La Fe, Valencia, Spain; 13 Centre Borelli, Université Paris-Saclay, ENS Paris-Saclay, CNRS, Service Desanté Des Armées, Université de Paris, Saclay, France

**Keywords:** Radiotherapy, Primary brain tumor, Cerebrovascular events, Stroke

## Abstract

**Purpose:**

Radiotherapy (RT) is a recognized risk factor for cerebrovascular (CV) disease in children and in adults with head and neck cancer. We aimed to investigate whether cerebral RT increases the risk of CV disease in adults with primary brain tumors (PBT).

**Methods:**

We retrospectively identified adults with a supratentorial PBT diagnosed between 1975 and 2006 and with at least 10 years follow-up after treatment. We analyzed demographic, clinical, and radiological features with special attention to CV events. We also described CV events, vascular risk factors, and intracranial artery modifications in a cross-sectional study of irradiated patients alive at the time of the study.

**Results:**

A total of 116 patients, treated with RT (exposed group), and 85 non-irradiated patients (unexposed group) were enrolled. Stroke was more frequent in irradiated PBT patients than in the unexposed group (42/116 (36%) vs 7/85 (8%); *p* < 0.001), with higher prevalence of both ischemic (27/116 (23%) vs 6/85 (7%); *p* = 0.004) and hemorrhagic (12/116 (10%) vs 1/85 (1%); *p* = 0.02) stroke. In the irradiated group, patients with tumors near the Willis Polygon were more likely to experience stroke (*p* < 0.016). Fourty-four alive irradiated patients were included in the cross-sectional study. In this subgroup, intracranial arterial stenosis was more prevalent (11/45, 24%) compared to general population (9%).

**Conclusions:**

Stroke prevalence is increased in long-surviving PBT patients treated with cranial RT.

**Implications for cancer survivors:**

CV events are frequent in long survivors of PBT treated with cerebral RT. We propose a check list to guide management of late CV complications in adults treated with RT for PBT.

**Supplementary Information:**

The online version contains supplementary material available at 10.1007/s11764-023-01350-z.

## Introduction

Radiotherapy (RT) plays a keyrole in the treatment of primary brain tumors (PBTs) [1]. However, it can induce several late complications such as leukoencephalopathy, radionecrosis, radiation-induced tumors, vascular diseases, or neurocognitive function [2–6].

Vascular complications include cavernous malformation, small-vessel disease, and stroke [5, 7, 8]. The pathophysiology of vasculopathy is unclear: it has been suggested that RT could accelerate atherosclerotic and/or induce inflammatory changes in large- and small-vessel arteries [9]. The resulting stenoses have already been shown to be responsible for an increased risk of ischemic cardiac events in long-term left breast cancer survivors receiving adjuvant radiotherapy [10], thus encouraging the development of specific coronary-sparing RT techniques [11, 12]. Recently, attention has been focused on small-vessel disease [13]. Endothelial cells are indeed supposed to be the most radiosensitive cells of the vessel wall, making small arteries and capillaries the vessels more vulnerable to radiation-induced damage [14, 15].

A higher prevalence of vascular events as a late complication of brain irradiation has been largely reported in pediatric populations [5, 16]. In adults, it is well established that RT for head and neck cancers increases the risk of stroke and transient ischemic attacks, due to large vessels damage [17, 18]. However, even if some studies about ischemic stroke in adults with PBT exist [19], the cerebrovascular consequences of brain RT for PBTs in adults are poorly studied.

In this paper, we sought to evaluate the risk of cerebrovascular (CV) events (ischemic or hemorrhagic) in adults treated with RT for PBTs. We performed a single-institution case–control study comparing PBT patients having received RT (exposed population) with PBT patients treated by chemotherapy only (non-exposed population). We further in-depth analyzed the characteristics of late-onset vascular complications performing a cross-sectional analysis including all patients in the exposed cohort that were alive at the time of the study.

## Materials and methods

### Case–control study

We performed a retrospective exposed-unexposed study from the institutional database of the Pitié-Salpêtrière Neuro-oncology department.We selected patients meeting the following inclusion criteria: (1) diagnosis of PBT between 1975 and 2006 (to allow sufficient follow-up time); (2) age ≥ 18 years at the time of diagnosis; and (3) at least 10-year clinical and radiological follow-up from diagnosis (in the non-irradiated group) or from radiotherapy (in the irradiated group). Both deceased and alive patients were included.

Patients diagnosed with primary central nervous system lymphoma or infratentorial tumors were excluded, as well as patients treated with craniospinal radiotherapy involving supra-aortic trunks.

Exposed patients were defined as those having been treated with RT to the brain while unexposed patients were those who had not received RT. All RT modalities were included, regardless of dose and schedule.

The clinical records of patients identified through this research were retrospectively reviewed for clinical and radiological data, with special attention to CV events, which were radiologically defined as follows:

- Acute ischemic stroke: a focal lesion corresponding to a large vessel or lacunar territory that is hyper-intense on diffusion-weighted magnetic resonance (MR) images and has a corresponding low value on the apparent diffusion coefficient map [20].
- Chronic ischemic stroke: a focal lesion corresponding to a large vessel or lacunar territory that is hypo-intense on T1-weighted MR images and hypo-intense with a hyper-intense boundary on fluid-attenuated inversion recovery (FLAIR) MR sequences, without corresponding changes on diffusion-weighted images [21].
- Hemorrhagic stroke: hypo-intense focal lesion on gradient recalled-echo T2*-weighted or susceptibility-weighted images [22].

We defined lacunar lesion those events localized in the vascular territories of the small penetrating arteries of the thalamus, gangliocapsular regions, corona radiata, and brainstem, which are a consequence of microvascular disease.

Conversely, large-vessel stroke corresponded to those localized in the territories of the anterior, middle, or posterior cerebral arteries.

Cardiovascular events directly attributable to the tumor growth or biological behavior (e.g., intratumoral hemorrhages, direct large-vessel compression) were excluded.

We also explored, in brain irradiated patients, whether radiotherapy was a risk factor of stroke when the treated tumor was situated near the Willis polygon (WP) defined as tumor located along the midline brain, or in the frontal and temporal lobes, at less than 2 cm from the circle of Willis. Since radiotherapy schedules were heterogenous, we could not exactly calculate the dose of radiation of the WP. Thus, 2 cm is an arbitrary cutoff distance beyond which we considered that the WP received low dose of radiation. The WP was defined as the intracranial internal carotid, middle cerebral, anterior cerebral, posterior cerebral, anterior communicating, posterior communicating, and basilar arteries.

### Cross-sectional study

All patients included in the exposed cohort that were alive at the time of the study were included in the cross-sectional analysis.

In our center, since 2014, every patient treated with brain radiotherapy systematically underwent a vascular study every 5 years, including a magnetic resonance angiography, and a laboratory blood test including HbA1c and lipidic panel. A Framingham score [23] was calculated to establish the vascular risk for each patient.

Brain MRIs were reviewed by a neurologist and a neuroradiologist for the presence of ischemic and/or hemorrhagic stroke. FLAIR MRI sequences were reviewed for leukoencephalopathy according to the Fazekas score, as follows: grade 0, no white matter change; grade 1, minimal patchy white matter foci; grade 2, start of confluence of white matter disease; grade 3, large confluent areas [24]. Brain atrophy was visually rated using the Global Cortical Atrophy Score, where 0 = none, 1 = mild, 2 = moderate, and 3 = severe atrophy [25]. Other signs of small-vessel disease such as perivascular spaces and cerebral microbleeds, as described in STRIVE v1 [13, 26, 27], were counted manually.

In patients with CV events, additional data were collected: age of the patient at the time of the event, stroke topography and etiology, clinical manifestations, and the delay from RT.

### Statistical analysis

Incidence of CV events in irradiated patients was displayed using cumulative incidence curves.

Time-to-event statistics were calculated using the Kaplan–Meier method and comparisons were made using a log-rank test. Patients characteristics were compared with the Wilcoxon or Student *t* test according to the sample size and data distribution for continuous variables and the chi-square test or Fisher’s exact test for categorical variables (according to the size of the sample). A *p* value of < 0.05 was considered significant. Statistical tests were performed using R Studio software (RStudio, PBC, Boston, MA).

## Results

**Fig. 1:**
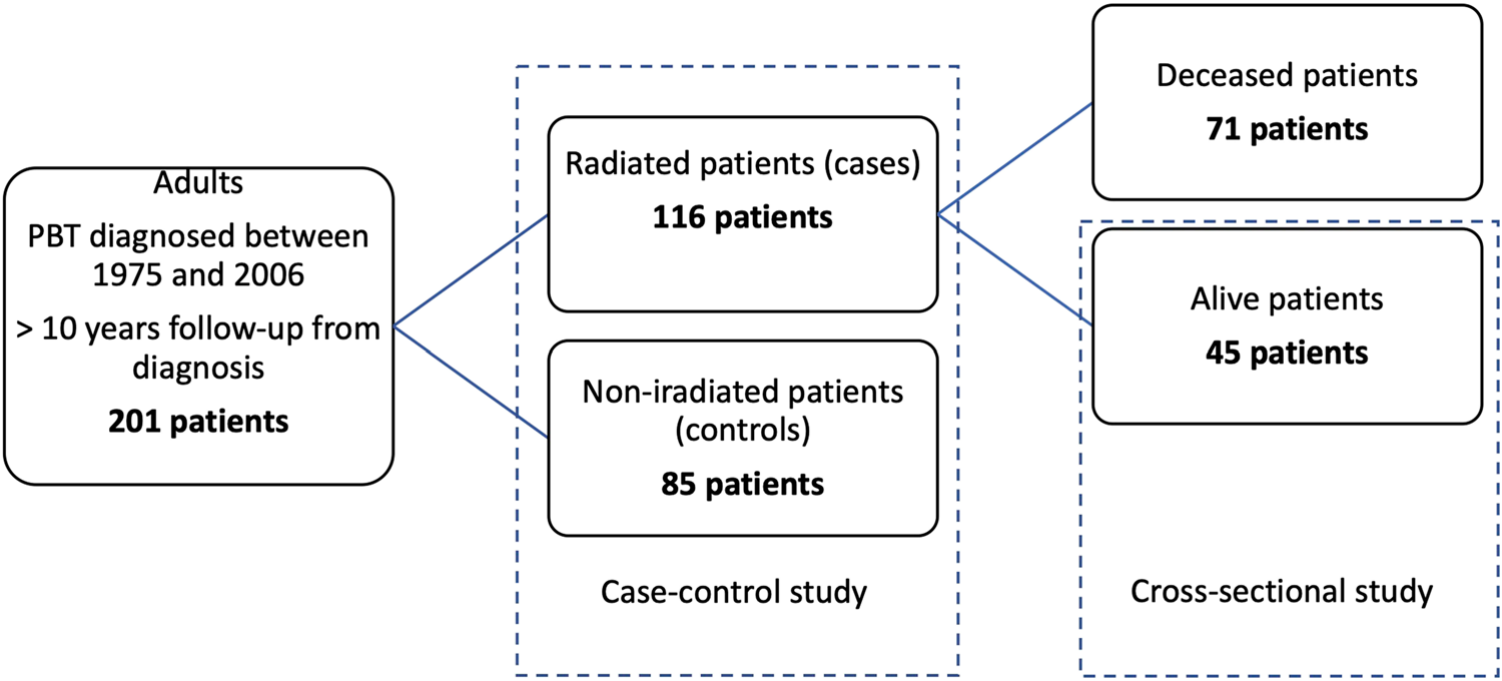
Study flowchart for patients and controls selection

A total of 201 PBT patients met the inclusion criteria, including 116 irradiated and 85 non-irradiated patients. Among the former, 45 were alive at the time of the study, and entered the cross-sectional analysis cohort (Fig. 1).

### Case–control study

All 201 patients were included in the exposed-unexposed case–control study. Clinical and demographical data are summarized in Table 1.

**Table 1:** Baseline characteristics in patients and controls. *NA* non-available. *Histology was not available in 3 cases (patients refused the surgery but were diagnosed as gliomas based on clinical and radiological features only and treated accordingly)

Variable	Irradiated group (*n* = 116)	Non-irradiated group (*n* = 85)	*p*-value
Mean age at diagnosis, years (range)	39.37 (18–62)	39.62 (18–70)	0.99
Mean age at RT, years (range)	41.41 (18–68)	NA	
Gender, *N* (%)			0.19
Male	67 (58%)	41 (48%)	
Female	49 (42%)	44 (52%)	
Surgery, *N* (%)			0.40
Biopsy	42 (36%)	22 (26%)	
Partial resection	35 (30%)	28 (33%)	
Complete resection	37 (32%)	34 (40%)	
No surgery	2 (2%)	1 (1%)	
Tumor histology, *N* (%)			0,22
Grade 2 astrocytoma	12 (10%)	16 (19%)	
Grade 3 astrocytoma	14 (12%)	0 (0%)	
Grade 2 Oligodendroglioma	21 (18%)	54 (62%)	
Grade 3 Oligodendroglioma	29 (25%)	4 (5%)	
Others	38 (33%)	11 (13%)	
NA*	2 (2%)	1 (1%)	
Tumor location, *N* (%)			
< 2 cm to Willis polygon	27 (33%)	NA	
> 2 cm from Willis polygon	89 (77%)	NA	
RT field, *N* (%)			
Focal	114 (98%)	NA	
Whole brain	2 (2%)	NA	

Histology distribution was different in the exposed and unexposed groups: there were more grade 2 glioma in the non-irradiated group (28.45% versus 82.35%, *p* < 0.0001), whereas grade 3 gliomas were more frequent in the irradiated group (37.07% versus 4.70%, *p* < 0.0001). Other demographic variables did not significantly differ among the two groups. Median follow-up time was 19 years for exposed patients and 14 years for the non-exposed group.

In the RT exposed group, all but two patients were treated with focal radiotherapy. Among them, the majority received 2D radiotherapy (56%), whereas 3D modality was used in 21% of cases.

The incidence of CV events was higher in irradiated (exposed) compared to non-irradiated (unexposed) patients (36%versus8%, *p* < 0.0001, Table 2).

**Table 2:** Cerebrovascular events in patients and controls. *N* number

	Exposed (irratiated)	Non-exposed (non-irradiated)	*p*-value
*N*	116	85	
Stroke, *N* (%)	42/116 (36%)	7/85 (8%)	< 0.0001
-Ischemic	27/116 (23%)	6/85 (7%)	0.004
-Hemorrhagic	12/116 (10%)	1/85 (1%)	0.02
-Ischemic and hemorrhagic	3/116 (3%)	0/116 (0%)	0.37
Vascular territory, *N* (%)			
-Large vessels	11/116 (10%)	1/85 (1%)	0.03
-Lacunar	19/116 (16%)	5/85 (6%)	0.04
Stroke location			
-Ipsilateral to tumor	25/116 (21%)	NA	
-Contralateral to tumor	17/116 (15%)	NA	

**Fig. 2:**
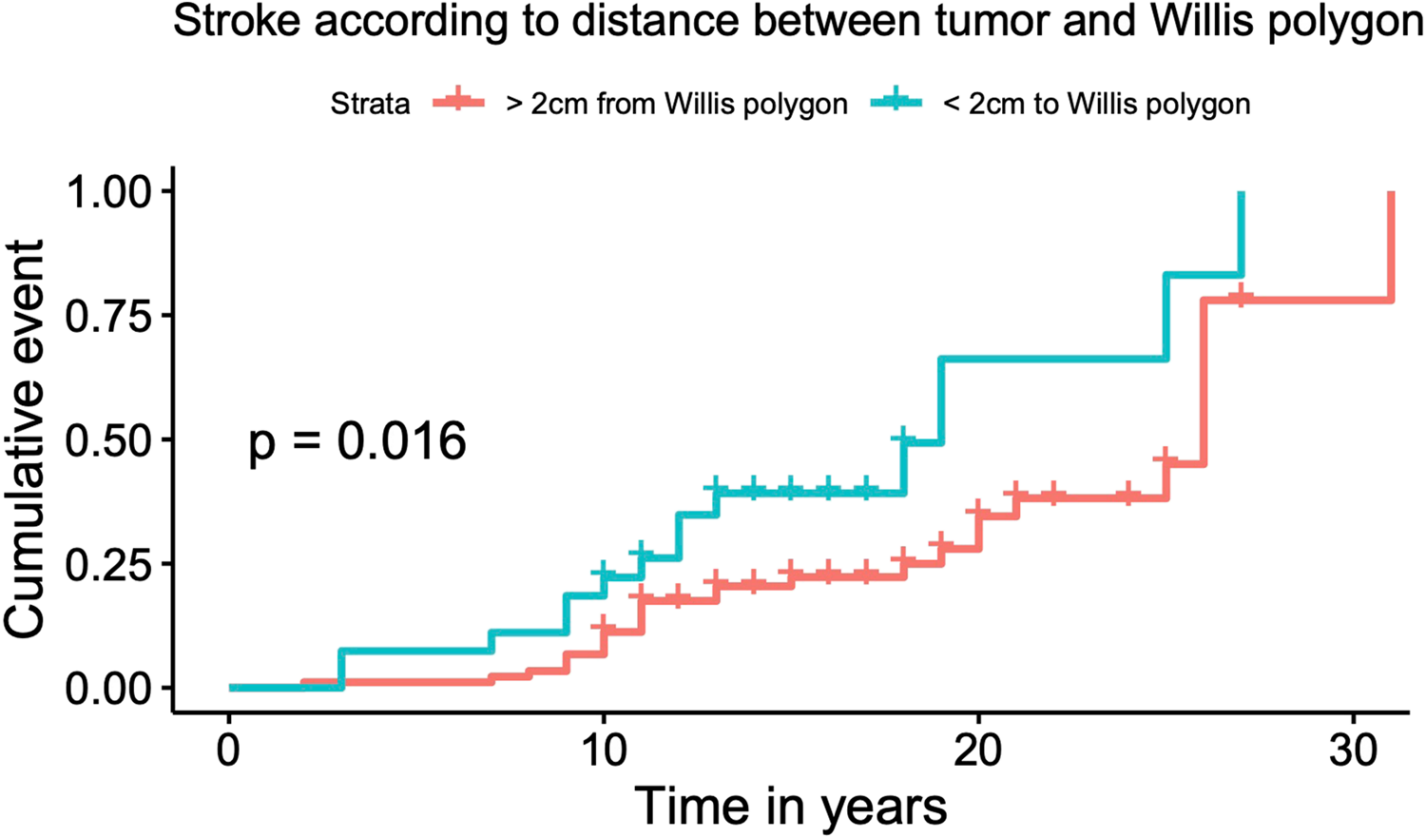
Incidence of CV events in irradiated patients according to distance between tumor and circle of Willis. Results were adjusted for sex and age

Among the 42 irradiated patients with a CV event, stroke was ischemic in 27 (64%), hemorrhagic in 12 (29%), and 3 (7%) patients had both ischemic and hemorrhagic events. In the non-exposed group, stroke was ischemic in 6 patients and hemorrhagic in one. Both ischemic and hemorrhagic events were more frequent in the irradiated group (*p* = 0.004 and 0.02 respectively, Table 2). In the irradiated group, both small- and large-vessel strokes were more frequent than in the nonirradiated group (*p* = 0.03 and 0.04 respectively, Table 2). Furthemore, in the irradiated group, among the 42 CV events, 25 (60%) were ipsilateral to their brain tumor. Among irradiated patients, CV events were more frequent when the treated tumor was located < 2 cm from the WP (14/27, 52% versus 28/89, 31%, *p* < 0.016) (Fig. 2).

### Cross-sectional study

The cross-sectional evaluation was performed in 45 previously irradiated patients that were alive at the time of the study. There were 27 males (60%) and 18 females (40%), with a median age at brain tumor diagnosis of 38 years (range 17–60 years) and at RT of 40 years old (range 18–68 years). Grade 3 oligodendroglioma was the most frequent histological diagnosis. Baseline data are available in Supplementary Table 1. Radiation features were quite homogenous, with a median total RT dose of 59.4 Gy in 31 sessions (range 22–46). Median follow-up time (from RT to the cross-sectional evaluation) was 18 years.

Twenty-five patients (55%) had a CV event, ischemic in 17 (68%) cases, and hemorrhagic in 5 (20%) cases. Three patients (12%) had both ischemic and hemorrhagic stroke. Ischemic stroke was symptomatic in 12 (71%) cases, with permanent sequelae in nine. In five cases, clinically silent ischemic stroke was diagnosed on a brain MRI performed during routine oncological follow-up. Median age at stroke was 55 years, with a median time of 12 years (range 2–31) from the end of RT. Among the three patients with both ischemic and hemorrhagic stroke, one presented with multiple cavernomas in the irradiated field. CV data of these 45 patients are available in Supplementary Table 2.

Among the 25 patients diagnosed with stroke, 19 had at least one vascular risk factor (high blood pressure, diabetes, cholesterol, smoke habit, alcoholism, or overweight). Atrial fibrillation was detected in 2 patients. Framingham risk score was calculated in each patient. No statistical difference was observed between patients who manifested with stroke and those who did not (median Framingham score 20 versus 16 respectively, *p* = 0.4).

Brain MRI angiography revealed intracranial arterial stenosis > 50% in 11 patients (11/45, 24%), which was symptomatic in five cases. Three patients had CV events in a different territory of the stenotic artery, and three others had no stroke at the time of study.

All other long-term radiological modifications observed in the cross-sectional study are detailed in Supplementary Table 3. Thirty-one patients (69%) had moderate to severe leukoencephalopathy. Thirty-three patients (73%) had moderate to severe cortical atrophy. Eighteen patients (40%) had 10 or more microbleeds, and 20 (44%) patients presented with vacuoles. Those modifications were similar in patients with and without CV event, since any statistical difference was found between the two groups. Representative images of long-term radiological modifications are shown in Supplementary Fig. 1.

## Discussion

We performed here a retrospective analysis on the long-term risk of CV events in adults treated with RT for PBTs. Among the 201 long-surviving patients included in this study, the exposure to brain RT was associated with an increased risk of both ischemic and hemorrhagic stroke. Patients treated for a tumor located near central arterial circulation (defined above as those involving the midline brain or situated in frontal or temporal lobes at less than 2 cm from the Willis polygon) seem more likely to experience stroke, as suggested by previous studies [5, 28]. This could be explained by the fact that large intracranial vessels would receive more important doses of radiation. Indeed, in the 45 patients included in the cross-sectional cohort, 11 (24%) had an intracranial stenosis in MRI angiography, by far more prevalent than what is reported for the general population (9%) [29]. These findings are in line with previous reports [30]. Given those findings, special attention must be paid when treating bening sellar or suprasellar tumors (i.e., craniopharyngiomas) situated in close proximity to the WP. Most patients are treated with surgery followed by high-dose RT. Recent studies have pointed out a higher prevalence of stroke in patients with craniopharyngioma treated with RT [31–33].

Nonetheless, lacunar lesions, which are believed to be a consequence of microvascular disease, appeared to be quite frequent in our study. Indeed, there are an increasing number of studies focusing on RT-induced small-vessel disease [8, 13]. Moreover, even if large-vessel events are deemed to be more symptomatic than small-vessel stroke, in our cross-sectional cohort, half of small-vessel events were symptomatic, with permanent sequelae in one-third of cases. This is relevant, as clinical deterioration due to small-vessel disease can be underappreciated or even misdiagnosed during the follow-up of PBT patients.

We have to point out the high prevalence of long-term radiological modifications observed in the cross-sectional study, such as leukoencephalopthy or brain atrophy. Even if those modifications are frequently reported to radiotherapy, the role of chemobrain is widely accepted too [34, 35].

Radiotherapy features are lacking in most of the patients of our series which is a weakness. We focused on long lasting survivors after PBT treatment and therefore enrolled patients irradiated long time away with RT technologies which are no longer used nowadays. We thus cannot assert any firm conlusion concerning RT modalities comparision. It is well accepted that the risk of late adverse events correlates with the volume of tissue irradiated and dose delivered. Conformal 3D RT aims to improve conformity of radiation to the target volume while sparing healthy brain and, consequently, reducing RT side effects. In the last years, more sophisticated techniques, such as intensity modulated RT (IMRT), are increasingly replacing 3D conformal radiotherapy, allowing a further reduction of off-target irradiation and, consequently, healthy brain toxicity [36–38].

Recent clinical practice guidelines for vascular complications of brain tumors suggest that the management depends on the underlying cause, the patient’s neurological condition, and the prognosis of the tumor [39]. Evidence-based guidelines for the management of radiation-induced vasculopathy are lacking. Medical community tends to consider that PBT patients with established vascular risk factors, such as hypertension, diabetes, dyslipidemia, or smoking, harbor a higher risk of CVevents after RT. Some studies have shown that hypertension and diabetes increased the stroke risk in previously irradiated patients [40–42]. However, those studies focused on pediatric population or in head and neck cancer patients and, to our knowledge, no data concerning brain irradiation in adults are available. In our series, no relationship was found between a high Framingham score and the incidence of stroke in previously irradiated patients. This may suggest that traditional vascular risk factors do not play a key role in the occurrence of stroke in this population. Nevertheless, since no other management strategies are available, we strongly recommend a strict control of vascular risk factors. Thus, we propose a simple check list, based on our personal experience, to guide the management of vascular complications in previously irradiated PBT patients (Table 3). In our opinion, a yearly cerebrovascular assessment should be undergone.

**Table 3:** Check list to guide management of vascular complications in long-surviving primary brain tumorspatients

1. Risk education/counseling
2. Blood pressure assessment
3. Laboratory test: lipidic profile, diabetes
4. Brain MRI ± angiography

The use of antiplatelet drugs for secondary prevention in previously irradiated brain tumor patients who experience an ischemic stroke is well accepted, even if dedicated studies in this population are lacking [43]. Conversely, the benefit of low-dose aspirin as primary prevention in patients who did not have an ischemic event remains unproven. In the absence of clinical or radiological evidence of stroke, this treatment is not recommended, also considering the non-negligible hemorrhagic risk in the tumor and the irradiated brain. Indeed, microbleeds are a frequent abnormality in brain MRI of long PBT survivors. The potential risks and benefits must be carefully evaluated before antiplatelet drug introduction. A recent study of Addison et al. reported that incidental statin use at the time of RT for head and neck cancer would be associated with a lower risk of stroke or TIA [44]. Further studies to evaluate the benefit of using statins and/or antiplatelet drugs in adult patients treated with RT for PBT are needed.

This retrospective study has several limitations which have to be pointed out. Several details, such as irradiation fields, could not be retrieved for a substantial part of patients, and were excluded from the current analysis. We therefore used tumor location to estimate the radiation of the WP, which is an approximative method. The arbitrary choice of a 2-cm cutoff distance from tumor to WP is discussed in an ad hoc limitations section. Information relating to vascular risk factors was not available for most of the patients in the case–control cohort. Thus, relationship between vascular risk factors and CV event was only studied in the cross-sectional study. Stroke dating was complicated in some cases, because of long periods of time between available brain MRI. Particularly, in the non-irradiated group, stroke dating was difficult for most patients. Thus, we could not perform a Kaplan-Meyer curve comparing CV events in exposed and unexposed patients. Furthermore, exposed and unexposed patients were not matched by vascular risk factors. The use of a larger sample and a longitudinal design would improve future studies. Finally, as already stated, RT techniques are continuously evolving, and newer modalities as IMRT are likely to further reduce the burden of radiation-induced adverse events, including vascular events.

Nonetheless, thanks to its long-term follow-up and its sample size, this study provides significant insight into the late risk of cerebrovascular events in patients receiving radiation as adults. Our study confirms an increased late risk of CV events, both ischemic and hemorrhagic, in long-surviving PBT, and especially in those with tumors located in close proximity to the Willis polygon.

Clinical and radiological follow-up of previously irradiated PBT patients should include yearly cerebrovascular assessment to individualize primary and secondary prevention strategies.

### Limitations

Since we did not have any information concerning the contouring process, we used tumor location to estimate the radiation of the WP, with an arbitrary cutoff distance of 2 cm from tumor to WP, which is an approximative method that could not be accurate. Current guidelines for diffuse and high-grade gliomas management recommend irradiating the gross tumor volume (GTV) and adding a margin of 1–2 cm to create the clinical target volume (CTV), and a supplementary margin of 0.3–0.5 mm to enable for uncertainties in patient setup and treatment delivery, generating thee planning target volume (PTV) [1]. The steep radiation dose fall-off in adjacent tissues is less important with old RT techniques, specially with 2D RT. The arrival of 3D conformational RT and more recently IMRT allow sharper radiation dose fall-off and thus the sparing of healthy brain tissue. Given that PTV includes the GTV and a supplementary margin of 1.5 to 3 cm, WP is likely to be included in the PTV when situated 2 cm from the tumor, specially when 2D RT has been used. Moreover, the fact that patients were treated with different RT techniques over a very long period introduces a chronological bias.

For all those reasons, it is important to point out that the results must be interpreted with caution and a 2-cm cutoff could not be considered a safety threshold.

### Supplementary Information

Below is the link to the electronic supplementary material.

Supplementary file1 (DOCX 12.6 KB)

Supplementary file2 (DOCX 12.2 KB)

Supplementary file3 (DOCX 13.1 KB)

Supplementary file4 (DOCX 85.0 KB)

## Data Availability

The datasets generated during and/or analyzed during the current study are available from the corresponding author on reasonable request.
