# Supplementary material for: Late-onset vascular complications of radiotherapy for primary brain tumors: a case–control and cross-sectional analysis
Source: J Cancer Surviv. 2023 May 5;18(1):59–67. doi: 10.1007/s11764-023-01350-z (PMC10867030; doi:10.1007/s11764-023-01350-z)
Supplement: Supplementary file 1 — Supplementary file1 (DOCX 12.6 KB) [file 11764_2023_1350_MOESM1_ESM.docx]

Supplementary Table 1: baseline characteristics of previously irradiated PBT patients included in the cross-sectional analysis

| **Variable** |  |
| --- | --- |
| **Numberof patients, N** | 45 |
| **Median age at diagnosis, years (range)** | 38 (18-60) |
| **Median age at RT, years (range)** | 40 (18-68) |
| **Gender, N (%)** |  |
| Male | 27 (60%) |
| Female | 18 (40%) |
| **Surgery, N (%)** |  |
| Biopsy | 10 (22%) |
| Partial resection | 17 (38%) |
| Complete resection | 17 (38%) |
| No surgery | 1 (2%) |
| **Histology, N (%)** |  |
| Grade 2 Astrocytoma | 3 (7%) |
| Grade 3 Astrocytoma | 4 (9%) |
| Grade 2 Oligodendroglioma | 5 (11%) |
| Grade 3 Oligodendroglioma | 17 (38%) |
| Other | 15 (33%) |
| No histology | 1(2%) |
| **Location, N (%)** |  |
| <2cm to Willis polygon | 11 (24.5%) |
| >2cm from Willis polygon | 34 (75.5%) |
| **Radiotherapy field, N** |  |
| Focal | 44 |
| Total dose received:   - Mean (range) - Median (IQR) | 56,92 (50-60)  59,4 (54-60) |
| Number of RT sessions:   - Mean (range) - Median (IQR) | 31,36 (22-46)  31 (30-33) |
