# Supplementary material for: Late-onset vascular complications of radiotherapy for primary brain tumors: a case–control and cross-sectional analysis
Source: J Cancer Surviv. 2023 May 5;18(1):59–67. doi: 10.1007/s11764-023-01350-z (PMC10867030; doi:10.1007/s11764-023-01350-z)
Supplement: Supplementary file 2 — Supplementary file2 (DOCX 12.2 KB) [file 11764_2023_1350_MOESM2_ESM.docx]

Supplementary Table 2: characteristics of stroke in affected patients in the cross-sectional cohort

| **Variable** |  |
| --- | --- |
| **Number of patients, N** | 25 |
| **Median age at stroke,** years (range) | 55 (37-78) |
| **Median time between RT and stroke**, years (range) | 12 (2-31) |
| **Stroke, N (%)** |  |
| - Ischemic | 17 (68%) |
| - Hemorrhagic | 5 (20%) |
| - Ischemic and hemorrhagic | 3 (12%) |
| **Ischemic stroke, N (%)** |  |
| - Lacunar | 12 (60%) |
| - Large vessel | 8 (40%) |
| **Ischemic stroke (symptoms), N (%)** |  |
| - Symptomatic | 13 (65%) |
| - Asymptomatic | 7 (35%) |
| - Sequelae | 9 (45%) |
| - No sequelae | 11 (55%) |
