# Supplementary material for: Late-onset vascular complications of radiotherapy for primary brain tumors: a case–control and cross-sectional analysis
Source: J Cancer Surviv. 2023 May 5;18(1):59–67. doi: 10.1007/s11764-023-01350-z (PMC10867030; doi:10.1007/s11764-023-01350-z)
Supplement: Supplementary file 3 — Supplementary file3 (DOCX 13.1 KB) [file 11764_2023_1350_MOESM3_ESM.docx]

Supplementary Table 3: other radiation-induced MRI changes and their association with the incidence of stroke

| **Variable** | **N (%)** | **P-value** | **OR** | **CI** |
| --- | --- | --- | --- | --- |
| **Leukoencephalopathy** |  | 0.17 | 2.533 | 0.669 9.585 |
| - Mild | 12 (27%) |  |  |  |
| - Moderate | 23 (51%) |  |  |  |
| - Severe | 9 (20%) |  |  |  |
| - None | 1 (2%) |  |  |  |
| **Brain Atrophy** |  | 0.64 | 1.067 | 0.255 4.463 |
| - Mild | 8 (18%) |  |  |  |
| - Moderate | 21 (47%) |  |  |  |
| - Severe | 13 (29%) |  |  |  |
| - None | 3 (6%) |  |  |  |
| **Vacuoles** |  | 0.20 | 2.195 | 0.648 7.436 |
| - No | 24 (53%) |  |  |  |
| - Yes | 21 (47%) |  |  |  |
| **Bleeds** |  | 0.64 | 0.111 | 0.007 1.776 |
| - < 10 | 22 (49%) |  |  |  |
| - 10 to 20 | 5 (11%) |  |  |  |
| - 20 to 50 | 8 (18%) |  |  |  |
| - > 50 | 4 (9%) |  |  |  |
| - None | 3 (6%) |  |  |  |
| - NA | 3 (6%) |  |  |  |
