# Supplementary material for: Late-onset vascular complications of radiotherapy for primary brain tumors: a case–control and cross-sectional analysis
Source: J Cancer Surviv. 2023 May 5;18(1):59–67. doi: 10.1007/s11764-023-01350-z (PMC10867030; doi:10.1007/s11764-023-01350-z)

Supplementary figure 1: long term radiological modifications in 4 patients.

Leucopathy on FLAIR images with focal brain atrophy (enlarged ventricule): arrowheads on A,B.

Enlarged perivascular spaces: white arrows in B, C, D, E.

Evolutive diffuse brain atrophy14 years (D) and 19 years (E) after radiotherapy.

Microbleeds(black arrows) and macrobleed in susceptibility-weighted image (F).


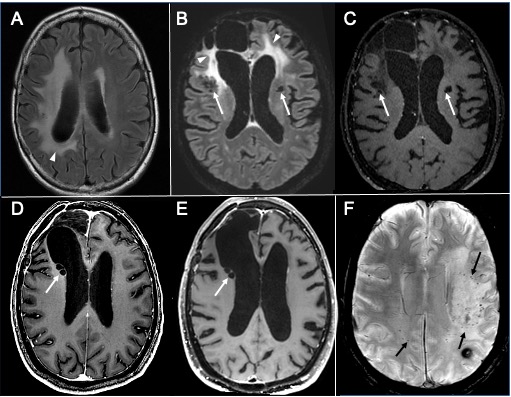

Supplement: Supplementary file 4 — Supplementary file4 (DOCX 85.0 KB) [file 11764_2023_1350_MOESM4_ESM.docx]
